# Transcriptomics and Alternative Splicing Analyses Reveal Large Differences between Maize Lines B73 and Mo17 in Response to Aphid *Rhopalosiphum padi* Infestation

**DOI:** 10.3389/fpls.2017.01738

**Published:** 2017-10-10

**Authors:** Juan Song, Hui Liu, Huifu Zhuang, Chunxia Zhao, Yuxing Xu, Shibo Wu, Jinfeng Qi, Jing Li, Christian Hettenhausen, Jianqiang Wu

**Affiliations:** ^1^Yunnan Key Laboratory for Wild Plant Resources, Department of Economic Plants and Biotechnology, Kunming Institute of Botany, Chinese Academy of Sciences, Kunming, China; ^2^University of the Chinese Academy of Sciences, Beijing, China; ^3^Dalian Institute of Chemical Physics, Chinese Academy of Science, Dalian, China; ^4^Yunnan Academy of Science and Technology Development, Kunming, China

**Keywords:** maize (*Zea mays* L.), *Rhopalosiphum padi*, transcriptome, alternative splicing, metabolites, benzoxazinoid

## Abstract

Maize (*Zea mays* L.) is a staple crop worldwide with extensive genetic variations. Various insects attack maize plants causing large yield loss. Here, we investigated the responses of maize B73, a susceptible line, and Mo17, a resistant line, to the aphid *Rhopalosiphum padi* on metabolite and transcriptome levels. *R. padi* feeding had no effect on the levels of the defensive metabolites benzoxazinoids (Bxs) in either line, and Mo17 contained substantially greater levels of Bxs than did B73. Profiling of the differentially expressed genes revealed that B73 and Mo17 responded to *R. padi* infestation specifically, and importantly, these two lines showed large gene expression differences even without *R. padi* herbivory. Correlation analysis identified four transcription factors (TFs) that might account for the high Bx levels in Mo17. Similarly, genome-wide alternative splicing (AS) analyses indicated that both B73 and Mo17 had temporally specific responses to *R. padi* infestation, and these two lines also exhibited large differences of AS regulation under normal condition, and 340 genes, including 10 TFs, were constantly differentially spliced. This study provides large-scale resource datasets for further studies on the mechanisms underlying maize-aphid interactions, and highlights the phenotypic divergence in defense against aphids among maize varieties.

## Significance statement

This is a comprehensive study on the defense responses of maize lines B73 and Mo17 to aphid herbivory. Aphid *Rhopalosiphum padi* infestation-induced defense-related metabolites and genome-wide changes of gene expressions and alternative splicing (AS) patterns were investigated, and we show that different maize lines have distinct defense-related responses to *R. padi* herbivory. This large-scale comparison between B73 and Mo17 provides key resource information and candidate genes for further identification of the regulatory network underlying maize defense against aphids.

## Introduction

Aphids (Hemiptera: Aphididae) are the most common phloem-feeding pests worldwide. They are devastating to host plants, including crops, due to their capability of very rapid population growth, deprivation of host photosynthates (carbon) and amino acids (nitrogen), reducing photosynthesis, and transmitting viruses (Hales et al., 1997; Smith and Boyko, 2007). The symptoms of aphid-attacked plants are diverse, such as chlorosis, necrosis, wilting, stunted growth, leaf curling, and gall formation (Guerrieri and Digilio, 2008), and vary according to aphid and plant species (Cooper and Goggin, 2005; Goggin, 2007).

Maize (*Zea mays*) is one of the most important crops as it is food to a large portion of the world population and is also used for animal feed and industry (Hu and Xu, 2011; Waisi et al., 2015). Globally, plant pests cause about 20–40% annual crop production losses, including maize (Oerke, 2005; FAO, 2017). However, how maize responds to insect herbivory, including aphids, is still not fully understood. Benzoxazinoids (Bxs), also known as cyclic hydroxamic acids, are important direct defense metabolites in certain poaceous plants and were first reported in maize and wheat (*Triticum aestivum* L.) (Wahlroos and Virtanen, 1959). Using maize plants having a mutation in the Bx biosynthesis gene *BENZOXAZINELESS1* (*BX1*), Ahmad et al. (2011) demonstrated that Bxs are very important metabolites in maize defense against *R. maidis*. Later, a genetic study further pointed to the toxicity of HDMBOA-Glc (2-hydroxy-4,7-dimethoxy-1,4-benzoxazin-3-one glucoside) to *R. maidis* and the callose inducibility of DIMBOA-Glc (2,4-dihydroxy-7-methoxy-1,4-benzoxazin-3-one glucoside) (Meihls et al., 2013).

In addition to compounds that are repellent, anti-nutritive, or toxic to insects, which are named direct defenses (Glauser et al., 2011; Handrick et al., 2016), plants also produce volatiles after herbivory, and some of these are important foraging cues for natural enemies of herbivores. These volatiles function as plant indirect defenses (Heil, 2008; de Vos and Jander, 2010). For example, during beet army worm (*Spodoptera exigua*) feeding, maize seedlings can perceive the elicitor volicitin in the caterpillar oral secretions and activate the biosynthesis of terpene volatiles, which attract herbivore parasitoids (e.g., *Cotesia marginiventris*) (Alborn et al., 1997; Schnee et al., 2006). Similarly, *Spodoptera littoralis*-attacked maize seedlings emit mono- and sesquiterpenes, which help parasitic wasps locate the larvae (Schnee et al., 2002). Insect oviposition can also elicit terpene emission in maize and the parasitic wasps are attracted to the oviposited maize (Tamiru et al., 2011, 2017).

Transcriptomic analyses have revealed changes in the expression levels of various genes in aphid-attacked plants (Thompson and Goggin, 2006). For example, Appel et al. (2014) demonstrated that feeding of green peach aphid *Myzus persicae* and cabbage aphid *Brevicoryne brassicae* up- and down-regulated various genes in *Arabidopsis*, but these genes were only 4–8% overlapping. In response to the corn leaf aphid (*Rhopalosiphum maidis*) infestation, maize line B73 showed dynamic transcriptomic changes, and the expression levels of hundreds of genes were altered at different time points investigated, with most of the genes up-regulated; further functional analyses revealed that most of the up-regulated genes were defense-related, while a number of down-regulated transcripts were related to primary metabolism (Tzin et al., 2015a). Another microarray study revealed that *M. persicae* infestation on *Arabidopsis* resulted in the largest number of changes in gene expressions, compared with those induced by pathogens *Pseudomonas syringae* and *Alternaria brassicicola*, chewing caterpillar *Pieris rapae*, and cell content-feeding thrips *Frankliniella occidentalis* (De Vos et al., 2005).

Alternative splicing (AS) is an important post-transcriptional regulatory mechanism in eukaryotes that processes premature mRNA into multiple mature isoforms, thus affecting mRNA stability and increasing transcriptome complexity and protein diversity (Black, 2003; Simpson et al., 2008). AS is involved in plant growth and development (Romanowski and Yanovsky, 2015; Srinivasan et al., 2016; Wang et al., 2017) and adaptation to abiotic and biotic stresses (Schliebner et al., 2014; Ren et al., 2015; Keller et al., 2016; Thatcher et al., 2016; Jiang et al., 2017). Recently, in response to the lepidopteran chewing insect *Manduca sexta*, 180 and 356 differentially spliced genes were identified in the roots and leaves of the wild tobacco *Nicotiana attenuata*, respectively, suggesting a role of AS in regulating plant defense against lepidopteran insects (Ling et al., 2015). However, whether and how AS is involved in plant resistance to aphids was unknown.

Many studies have revealed very large genetic diversity in maize (Buckler et al., 2006), leading to broad variations in its resistance to insect herbivores (Meihls et al., 2012; Tzin et al., 2015b). For example, *R. maidis* produced about 20 times more progeny on the inbred line B73 than on Mo17 (Betsiashvili et al., 2015), and six commonly grown maize hybrids in Iran, namely K3640/3 × MO17, Simon, SC704, EXP1, VRE26 × K18, and VRE27 × K18, showed large differences in resistance to *R. maidis* (Razmjou and Golizadeh, 2010). In this study, we used mass spectrometry-based metabolite and Illumina RNA-seq analyses to investigate the defense responses to *R. padi*, a commonly occurring insect pest in maize, in two different maize inbred lines, the resistant line Mo17 and the susceptible line B73. Our data reveal extensive differences between B73 and Mo17 in their responses to *R. padi* feeding on metabolite and transcriptome levels, including gene expression and AS patterns. These data provide new insight into the regulatory elements that might shape the differences between B73 and Mo17.

## Materials and methods

### Plant growth and aphid rearing

Seeds of maize inbred lines B73, Zong3, Chang7-2, A188, and Mo17 were sown in 12 cm-diameter plastic pots filled with humus soil and vermiculite (about 7 to 1 ratio). Plants were grown in a greenhouse at ~17°C (night) and ~28°C (day) under a 14 h photoperiod until the fourth leaf had fully expanded (V4, 3-week-old seedlings). A *R. padi* colony was established from a single first instar nymph collected from a field in the Kunming Institute of Botany, Chinese Academy of Sciences, in Kunming, China, in 2014. The insect colony was maintained in an 80 × 80 × 150 cm cage on sorghum plants at 17–28°C, 14 h day length.

### Aphid bioassays

To evaluate the survival rates of *R. padi*, 20 apterous adults were transferred to the middle of the forth leaves and enclosed in clip cages (3 cm diameter), and after 24 h, the adult aphids were brushed off and 20 neonates were left in the clip cages to evaluate every day in the next 5 days (20 replicated maize plants for each group) (Carena and Glogoza, 2004; Mao and Zeng, 2014). To determine days from birth to first reproduction, life span, and progeny per aphid, one new neonate was placed on the middle of the forth leaf of each plant and the performance parameters were estimated until its death (20 replicated maize plants for each group) (Kettles et al., 2013).

### Plant treatments and sample collection

For treatment on maize, aphid feeding and mock treatments were performed as the following: for each aphid-treated plant, 50 adult aphids were gently placed onto the middle of the fourth leaf and enclosed in a clip cage, and the plants of the control group (mock) were fixed with empty clip cages. Three replicates were used for the aphid feeding and control group. In order to minimize the variations caused by circadian rhythm and other differences in environmental conditions, we treated leaves at different time points prior to the collection time. Aphids were quickly removed with a soft paintbrush at the time of harvest, and the regions in the cages were immediately excised and snap-frozen in liquid nitrogen.

### Metabolite data acquisition and analyses

50 adult aphids were feeding on plant for 0 and 48 h, five biological replicates were used, and a previously established method was employed (Qi et al., 2016). In brief, samples were extracted with 80% methanol and then analyzed on an Agilent 1200 Rapid Resolution Liquid Chromatography (RRLC) system equipped with a ZORBAX SB-Aq column (2.1 × 100 mm, 1.8 μm) followed by an Agilent 6510 Q-TOF running in positive ionization mode. The column temperature was set at 50°C, and the flow rate was 0.3 mL min^−1^. All metabolites were identified based on pure standards, except some Bxs: DIMBOA was confirmed by comparing the Bx profiles between maize inbred line H88 and *bx1* mutant (GRMZM2G085381); DIMBOA-Glc, DIM_2_BOA-Glc, HDMBOA-Glc, and MBOA were confirmed with purified standards, and the other putative Bxs were identified by their molecular masses. The abundance of each metabolite was estimated from the peak area. Peak detection and matching were performed using bioconductor XCMS and CAMERA packages (http://www.bioconductor.org/.) Samples (metabolites after integration) with *P* < 0.05 were considered to be differentially regulated. The acquired data were introduced to the SIMCA-P software package (v11.5, Umetric, Umea, Sweden) for Principal Component Analysis (PCA). The data was filtered by orthogonal signal correction (OSC) to remove variations from non-correlated factors.

### RNA-seq data acquisition and analysis of differentially expressed genes

Three biological replicates were used for RNA-seq analyses. Total RNA was extracted from ground leaf samples using TRIzol reagent (Invitrogen). The concentration of total RNA was measured using a NanoDrop 2000 Spectrophotometer (Thermo Scientific). Sequencing was performed at 3.75 G depth on a HiSeq2500-PE125 platform (Illumina). The raw sequence data are deposited in NCBI's Short Read Archive under the BioProject ID (PRJNA338511). The resulting sequences were trimmed based on quality scores and mapped to the maize B73 reference genome sequence V2 and maize working gene set V5a with Tophat2 (Kim et al., 2013) using the following modifications from the default parameters: maximum intron size 100,000; minimum intron size 20; up to two mismatches allowed (Trapnell et al., 2012). The expression levels of genes were estimated using Cufflink and were normalized using the numbers of reads per kb of exon sequence in a gene per million mapped reads. Differential expression analysis was performed using the Cuffdiff (Trapnell et al., 2012) with a cutoff of 2-fold change relative to the control levels.

### Alternative splicing analysis

All AS analyses were based on splice junctions obtained from the BAM files produced by TOPHAT2. For differential splicing analysis, we used Cuffdiff to categorize individual transcripts based on their transcription start site (Sammeth et al., 2008), thus grouping all the transcripts with a common pre-mRNA molecule. Thereafter, we performed Cuffdiff to estimate significant expression differences of each transcript using the Jensen-Shannon divergence metric across the different samples (Trapnell et al., 2012).

### Functional annotation and classification

For gene ontology (GO) analysis, we obtained the GO terms for each maize gene using the AgriGO web service (Du et al., 2010), and WEGO (Ye et al., 2006) was used for GO functional annotation; Plant MetGenMAP (Joung et al., 2009) was used for pathway enrichment analysis. Sets of genes showing a similar pattern of transcription were assumed to be functionally correlated. Transcription patterns were clustered using MeV 4.9.0 (Saeed et al., 2003) with Euclidean distances and the hierarchical cluster method of complete linkage clustering and Java software. MeV 4.9.0 was also used to create heat-map visualizations of differentially expressed genes. To identify the transcription factors, we referenced a maize transcription factor database retrieved from the Plant Transcription Factor Database 4.0 (Jin et al., 2015, 2017).

## Results

### Determination of the resistance levels of different maize lines to *R. padi*

**Figure 1:**
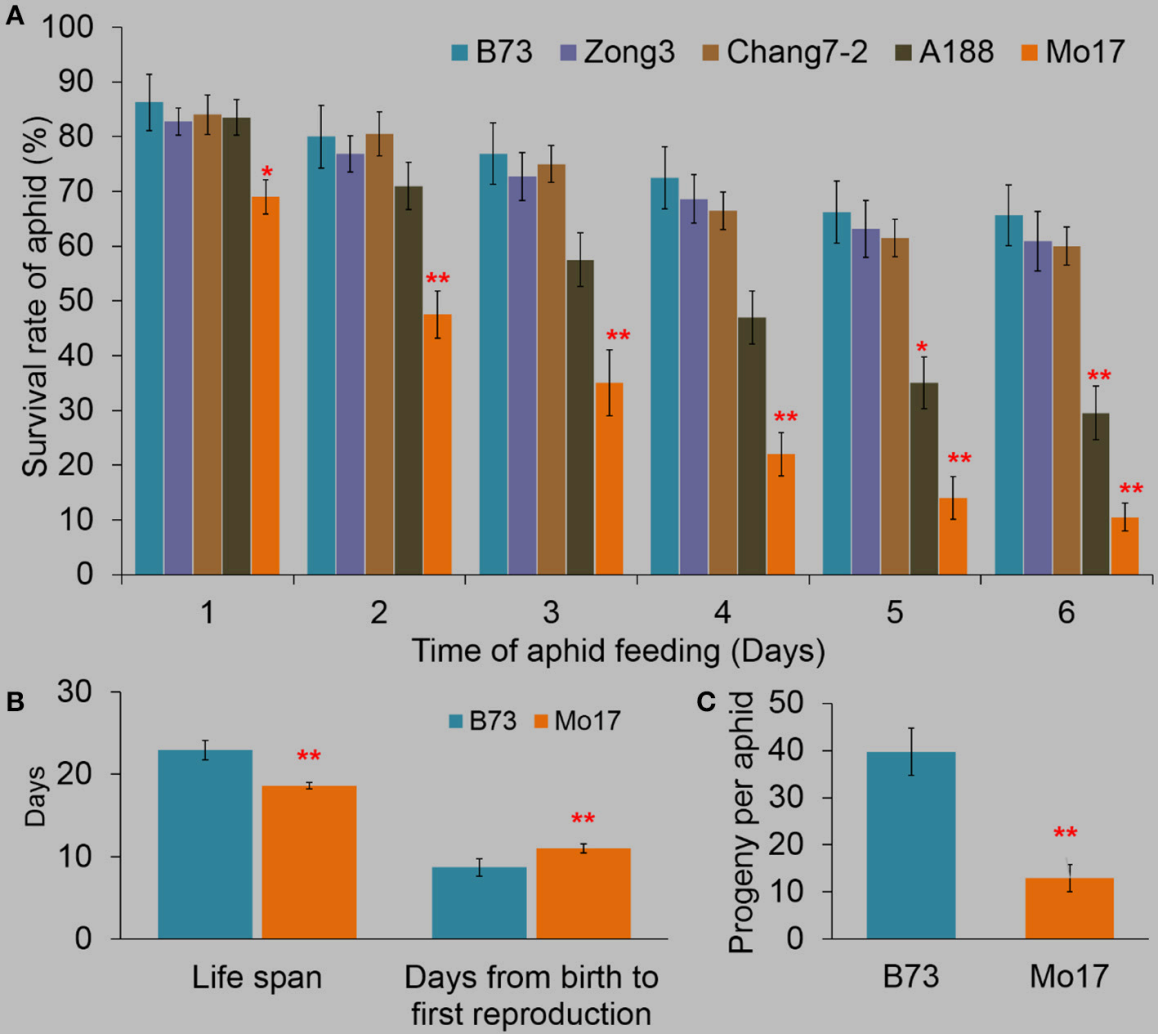
The resistance levels of different maize lines to *R. padi*. **(A)** The survival rates of *R. padi* on B73, Zong3, Chang7-2, A188, and Mo17. **(B)** The life span and the time needed from birth to first reproduction. **(C)** Fecundity of *R. padi* on B73 and Mo17. Values = means ± SE; *n* = 20; one-way ANOVA was used in **(A)**, *t*-test was applied in **(B,C)**; ^*^P < 0.05; ^**^P < 0.01).

To evaluate the levels of resistance to *R. padi* in different maize inbreed lines, we measured the survival rates of *R. padi* on the fourth fully expanded leaves of the inbred lines B73, Zong3, Chang7-2, A188, and Mo17 for 6 days (Figure 1A). *R. padi* showed the highest survival rate on B73 (86.3% survived on day 1), while exhibited the lowest survival rate on Mo17 (69% survived on day 1). The survival rates decreased in B73 and Mo17 and the trend continued in the next 5 days (65.6 and 10.5% aphids survived on B73 and Mo17 respectively on day 6) (Figure 1A). Congruently, the life spans of *R. padi* on B73 and Mo17 were 22.9 and 18.6 days, respectively, and the new neonates on B73 needed 8.7 days to start to give offspring, while 11 days were needed on Mo17 (Figure 1B). *R. padi* fecundity on B73 was much higher than on Mo17 (39.7 and 12.9 offspring per aphid on B73 and Mo17, respectively) (Figure 1C). Thus, B73 and Mo17 are a *R. padi*-susceptible and a-resistant line, respectively. These results were similar to previous findings, where Mo17 exhibited a much greater defense level against the maize leaf aphid *R. maidis* than did B73 (Meihls et al., 2013; Betsiashvili et al., 2015).

### Changes of metabolites after *R. padi* feeding

To explore the metabolites in B73 and Mo17 that might account for the observed *R. padi* resistance, leaf samples were collected 48 h after *R. padi* infestation. The profiles of Bx and phenolic metabolites were obtained on an UPLC-Q-TOF MS system (Table S1).

**Figure 2:**
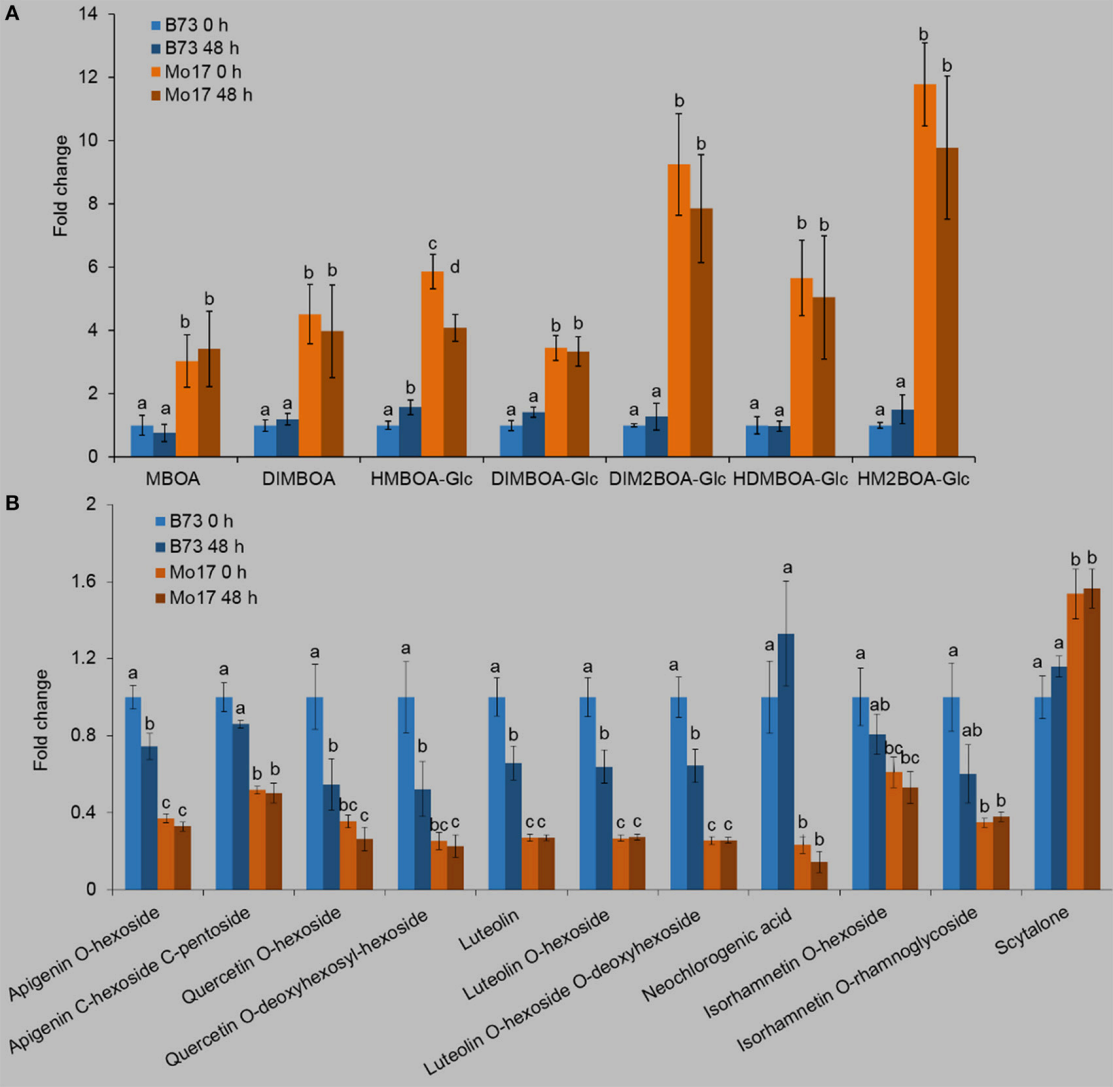
Profiles of Bxs and phenolics in B73 and Mo17. Maize leaves were treated with *R. padi* herbivory, and samples were collected at 48 h; non-treated leaves severed as controls (0 h). Relative contents of Bxs **(A)** and phenolics **(B)** in B73 and Mo17. Values = means ± SE; *n* = 5; different letters above bars indicate significant differences (one-way ANOVA and Duncan's multiple range test; *P* < 0.05); all values of the B73 group are normalized to 1. Complete data can be found in Table S1.

Given the importance of Bxs in maize defense against insects (Long et al., 1977; Rostas, 2007; Niemeyer, 2009; Glauser et al., 2011), we quantified the contents of Bxs (Figure 2A, Table S1). In general, Bxs were much higher in Mo17 than in B73. For example, DIMBOA, DIM_2_BOA, DIMBOA-Glc, and DIM_2_BOA-Glc in Mo17 were at least 2-fold > B73 (Figure 2A, Table S1). Notably, *R. padi* feeding did not have an obvious impact on the contents of Bxs in either line. Another group of metabolites that we examined were the phenolics. The contents of phenolics also showed large differences between B73 and Mo17. Without *R. padi* treatment, 10 phenolic compounds, apigenin O-hexoside, apigenin C-hexoside C-pentoside, quercetin O-hexoside, quercetin O-deoxyhexosyl-hexoside, luteolin, luteolin O-hexoside, luteolin O-hexoside O-deoxyhexoside, neochlorogenic acid, isorhamnetin O-hexoside, and isorhamnetin O-rhamnoglycoside were 0.64–3.29-fold higher in B73 than in Mo17, while the content of scytalone was 34.9% lower in B73 (Figure 2B, Table S1). After 48 h of *R. padi* feeding, most phenolics decreased in B73 (39–77%), but remained constant in Mo17.

### *R. padi* infestation-induced regulation of gene expressions in B73 and Mo17

We used RNA-seq technology to analyze the genome-wide gene expression responses of B73 and Mo17 to *R. padi* feeding at 6 and 24 h.

Differentially expressed genes (DEGs) were selected based on significance (*P* < 0.05) (Table S2): 3,789 and 3,420 DEGs were found in B73 after 6 and 24 h *R. padi* feeding, and the same treatment resulted in 4,127 and 1,786 DEGs in Mo17. Gene functions were further classified using GO analysis. As shown in Figure S1 (Table S2): 5,657 DEGs in B73 and 4,683 DEGs in Mo17 were assigned to 25 GO terms (Cellular Component, Molecular Function, and Biological Process: 8, 6, and 11 GO terms, respectively). The GO terms in B73 and Mo17 were largely similar except that some from Cellular Component (Extracellular Region Part and Macromolecular Complex) and Biological Process (such as Biological adhesion and Cellular Component Organization) showed significant differences.

**Figure 3:**
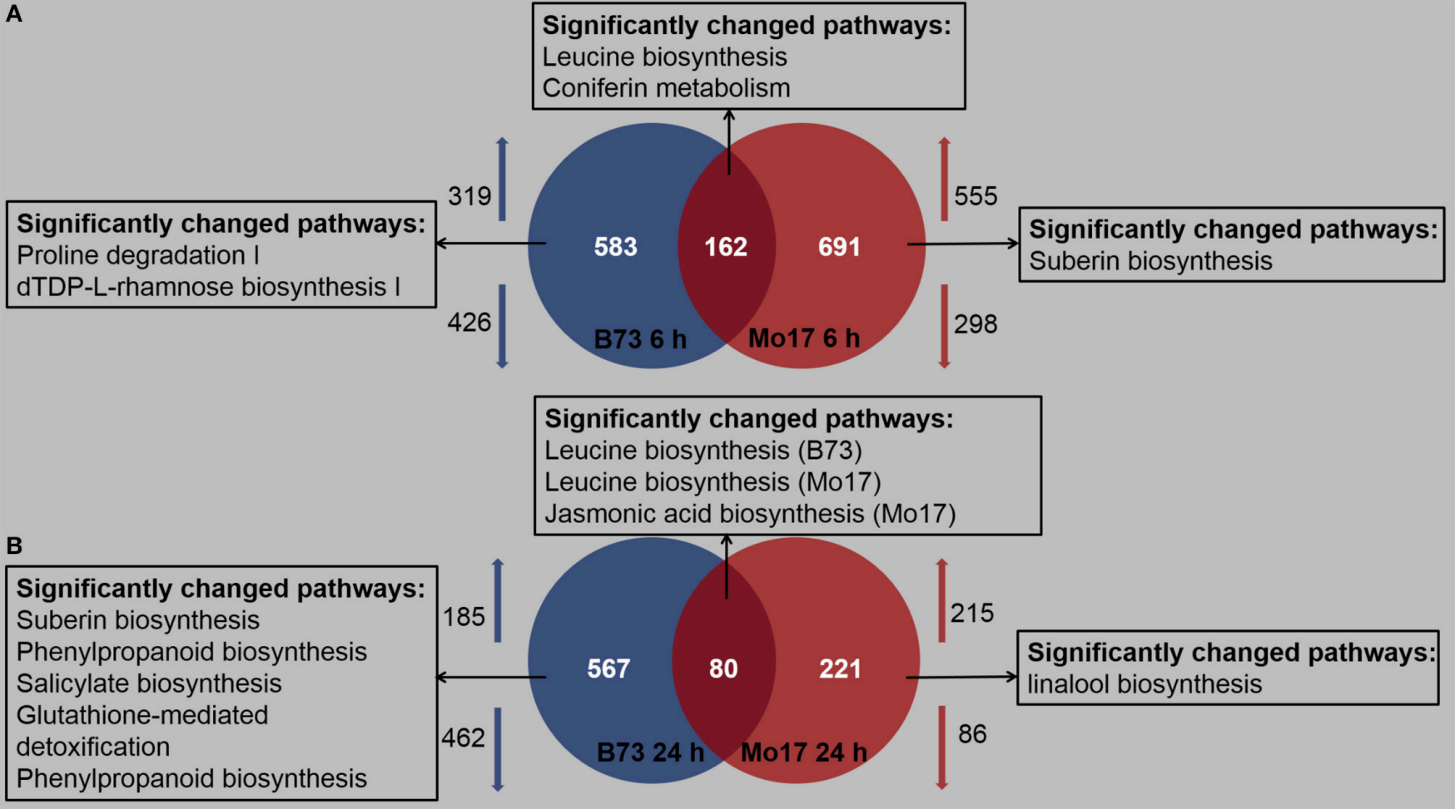
Profiles of regulated maize transcripts after 6 and 24 h of *R. padi* infestation on B73 and Mo17. The fourth maize leaves were each treated with 50 aphids, and samples were collected at 6 and 24 h; untreated leaves served as controls. Overall transcriptionally regulated genes and functional distribution of differentially expressed unique genes (fold change >2, *P* < 0.05) after 6 h **(A)** and 24 h **(B)** of *R. padi* herbivory in B73 and Mo17. Arrows indicate up- and down-regulated genes, respectively. Complete data can be found in Table S3.

Detailed expression profiling, based on the criteria that the genes should be up- or down-regulated more than 1-fold and *P* < 0.05, indicated that both B73 and Mo17 responded to *R. padi* infestation, but in a different manner (Table S3). After 6 h of *R. padi* feeding (Figure 3A, Table S3), 745 (319 up- and 426 down-regulated) and 853 (555 up- and 298 down-regulated) genes showed changes in expression levels in B73 and Mo17, respectively, and among these, only 162 were regulated in both lines. After 24 h of *R. padi* infestation (Figure 3B, Table S3), still 567 DEGs were uniquely regulated in B73, while Mo17 had specific 221 DEGs; only 80 genes showed altered levels in both B73 and Mo17 at 24 h. Thus, B73 and Mo17 have very different transcriptomic responses to *R. padi* infestation.

**Figure 4:**
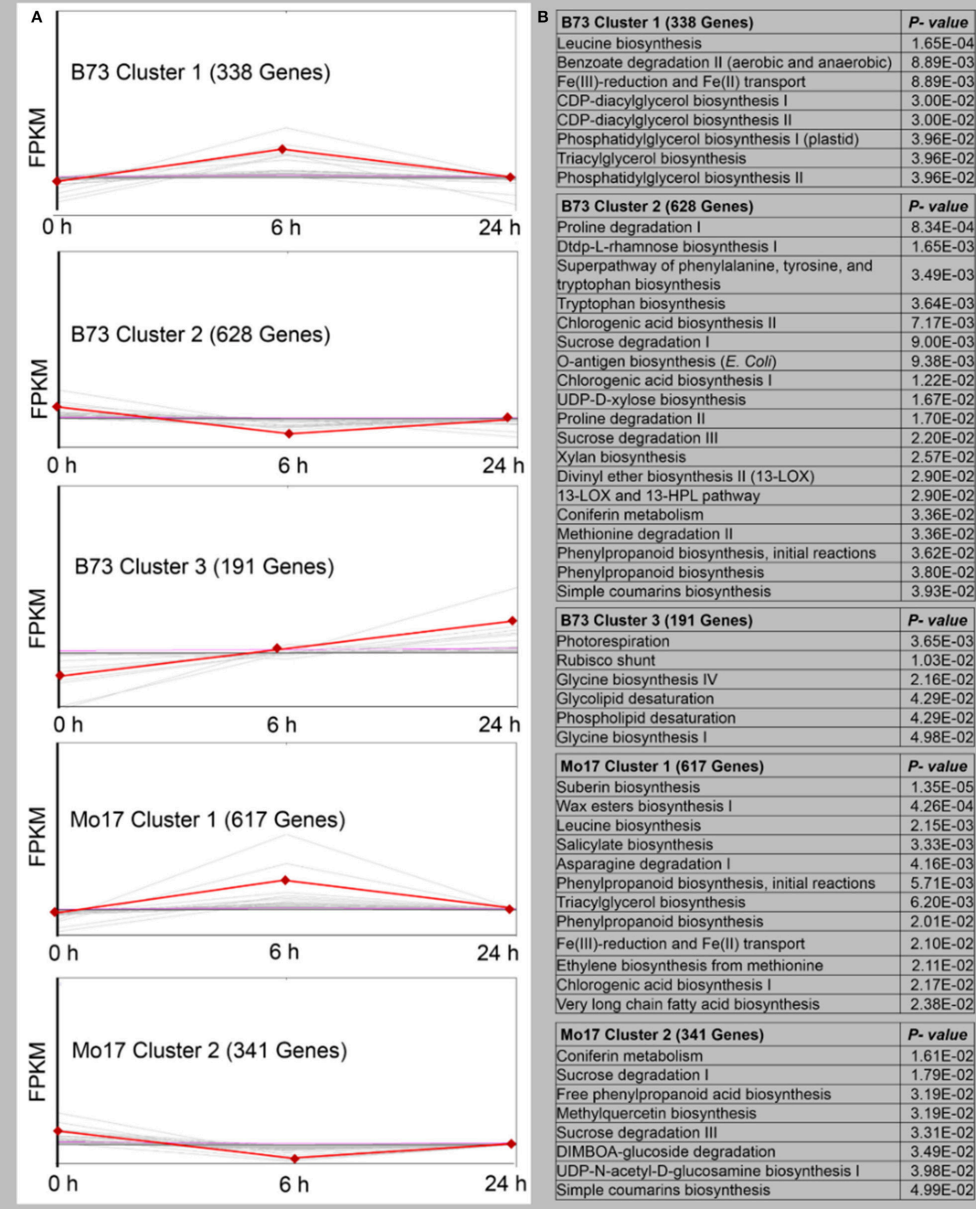
Overview of gene expression trends and clusters in *R. padi*-treated B73 and Mo17. **(A)** A total of 1157 transcripts in B73 (in three clusters) and 958 transcripts in Mo17 (in two clusters) with significant expression profile changes for at least one time-point after the *R. padi* feeding. The total number of transcripts in each cluster is indicated in the brackets, and the data for individual genes are shown in light gray. Average expression values for each cluster are shown in red. All genes selected for this analysis have significant differences of 1-fold (up- or down-regulated), *P* < 0.05. **(B)** Pathway enrichment analysis of each cluster using MetGenMAP to identify metabolic functions that were regulated. Full descriptions can be found in Table S4.

The DEGs were screened with a cluster analysis based on the k-means method using Pearson's correlation distance (TM4 software, http://www.tm4.org/). Each cluster represents genes whose expressions showed similar response patterns to *R. padi* infestation (Figure 4A, Table S4). In B73, 1,157 genes were classified into three expression cluster groups (Clusters 1–3). B73 Cluster 1, which contained 338 genes, showed a positive slope from 0 to 6 h, followed by a drop in the expression level at 24 h. The most abundant group was the B73 Cluster 2, with 628 genes showing a negative slope from 0 to 6 h, and then returned to the basal levels at the 24 h time point. B73 Cluster 3 had 191 genes whose expression showed a positive slope from 0 to 24 h. In Mo17, 958 transcripts were clustered into two groups (Figure 4A, Table S4). Cluster 1 contained 617 genes whose expression showed a positive slope from 0 to 6 h, followed by a drop in the expression level at 24 h, and Cluster 2 contained 341 genes whose expression showed an initial drop from 0 to 6 h, followed by reversion to the basal levels at 24 h (Figure 4A, Table S4). To reveal the different biological processes in each gene expression cluster, pathway enrichment analysis was performed using MetGenMAP (Joung et al., 2009). Pathways that were enriched among the transcripts in *R. padi*-treated B73 included (1) many plant defense- and stress response-related genes (Figure 4B, Table S4), such as benzoate degradation II in Cluster 1; (2) proline degradation, tryptophan biosynthesis, and chlorogenic acid biosynthesis I in Cluster 2; (3) photorespiration, Rubisco shunt, glycine biosynthesis, phospholipid desaturation, and glycolipid desaturation, which were mostly related to primary metabolism in Cluster 3. Notably, defense-related salicylate biosynthesis and chlorogenic acid biosynthesis could be found in Cluster 1, and DIMBOA-glucoside degradation was in Cluster 2 (Figure 4B, Table S4).

**Figure 5:**
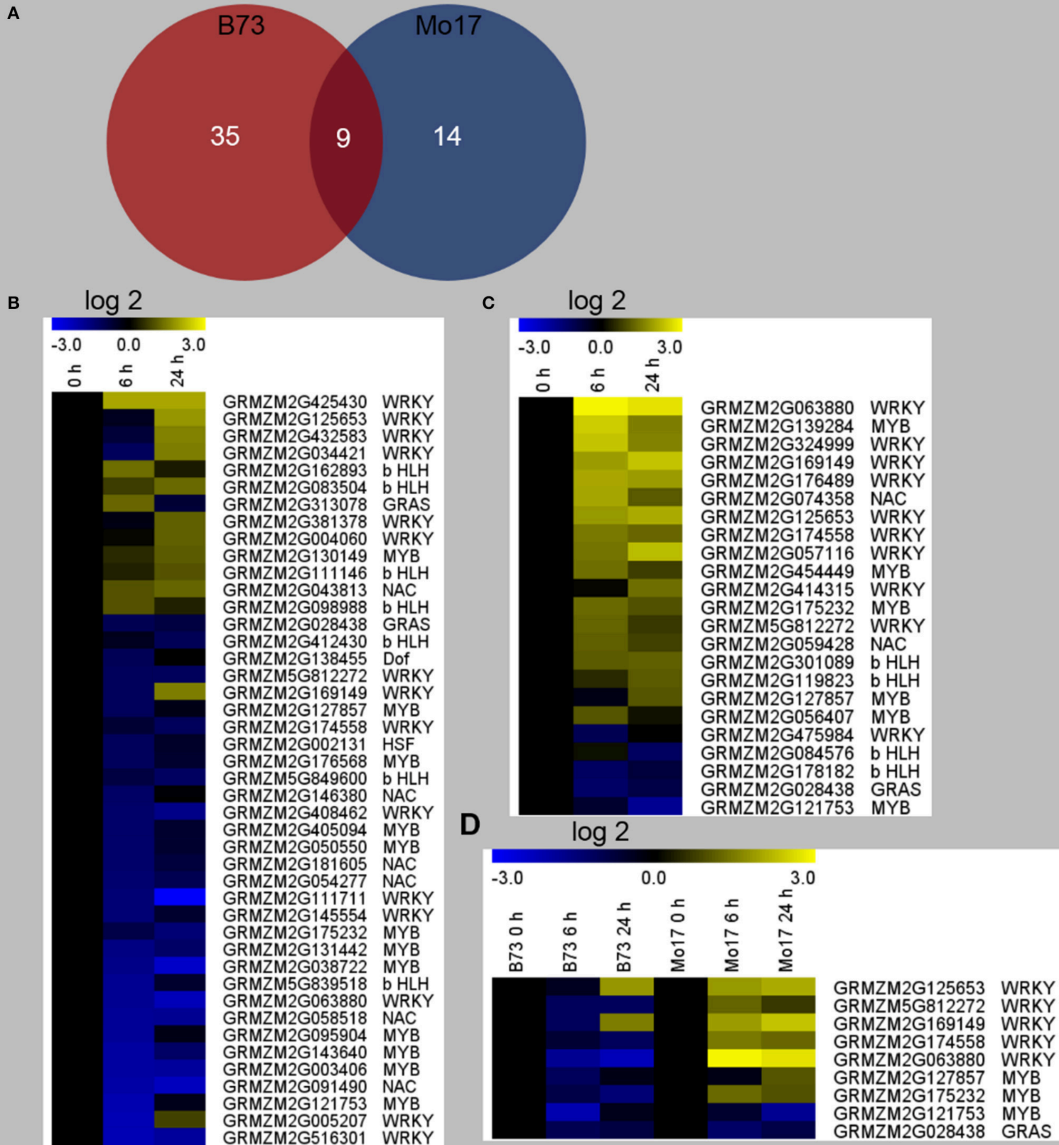
Differentially regulated transcription factors in B73 and Mo17 after *R. padi* herbivory. The fourth maize leaves were each treated with 50 aphids, and samples were collected at 6 and 24 h after *R. padi* treatment; untreated leaves served as controls. **(A)** Venn diagram depicting the number of transcription factors (TFs) significantly changed in B73 and Mo17 after 6 and 24 h of *R. padi* treatment. Heat maps indicate relative expression levels (fold change after log_2_ transformation) of TFs regulated by *R. padi* feeding in B73 **(B)** and Mo17 **(C)**. **(D)** Relative expression levels (fold change after log_2_ transformation) of the 9 common regulated TFs in B73 and Mo17. Detailed data can be found in Table S5.

In order to gain further insight into the regulatory mechanism underlying the differences between the responses of B73 and Mo17 to *R. padi* on the gene expression level, differentially regulated TFs were identified. It was found that *R. padi* herbivory specifically regulated 35 and 14 TFs in B73 and Mo17, respectively, and 9 TFs were regulated in common (Figure 5A, Table S5). We found that the 44 TFs distributed in seven families (MYB, WRKY, b HLH, NAC, GRAS, HSF, and Dof) in B73, most of which were down-regulated (Figure 5B, Table S5). In contrast, the 23 TFs in Mo17 distributed in five families (MYB, WRKY, b HLH, NAC, and GRAS), and most of them were up-regulated (Figure 5C, Table S5). However, 7 out of the 9 commonly regulated TFs in B73 and Mo17 were down-regulated in B73 but were up-regulated in Mo17 (Figure 5D, Table S5).

### Clustering of expression patterns in B73 and Mo17

Given the large gene expression differences between B73 and Mo17 in response to *R. padi* feeding, we expected that these two lines may also show a distinct transcript pattern at individual time points.

**Figure 6:**
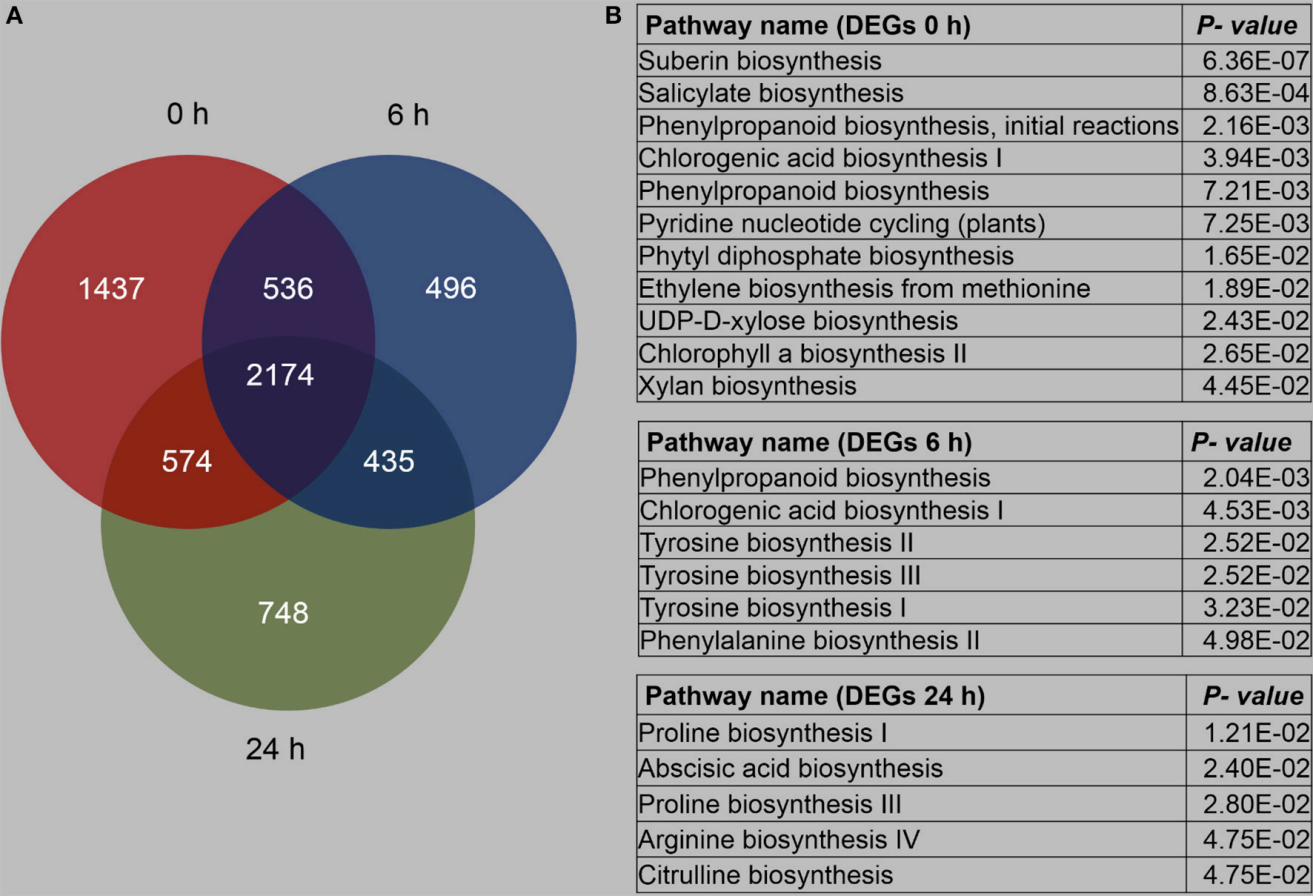
Overview of differentially expressed genes between B73 and Mo17. The fourth maize leaves were each treated with 50 aphids, and samples were collected at 6 and 24 h; untreated leaves served as controls (0 h). **(A)** Number of genes with differentially expressed levels between B73 and Mo17. **(B)** Pathway enrichment analysis using MetGenMAP to identify metabolic functions that were differentially expressed between B73 and Mo17. Full descriptions can be found in Table S6.

We compared the DEGs between B73 and Mo17 under the control condition and after 6 and 24 h of *R. padi* feeding, and it was found that 2,174 genes were always differentially expressed under all these conditions, but specifically, 1,437, 496, and 748 genes exhibited different expression levels only under control conditions and after 6 and 24 h of *R. padi* feeding, respectively (Figure 6A, Table S6).

Specifically, (1) the DEGs between B73 0 and Mo17 0 h were clustered to 11 pathways, most of which were associated with primary metabolism, except salicylate biosynthesis and chlorogenic acid biosynthesis I (Figure 6B, Table S6); (2) DEGs between B73 and Mo17 at 6 h were linked to four defense-related pathways, which were phenylpropanoid biosynthesis, chlorogenic acid biosynthesis I, tyrosine biosynthesis, and phenylalanine biosynthesis; (3) DEGs between B73 and Mo17 at 24 h were enriched on five pathways: Proline biosynthesis I, Abscisic acid biosynthesis, Proline biosynthesis III, Arginine biosynthesis IV, and Citrulline biosynthesis (Figure 6B, Table S6). Thus, B73 and Mo17 showed different response to *R. padi* feeding in gene expression pattern. More detailed transcriptomic comparisons between B73 and Mo17 over time are shown in Table S6.

**Figure 7:**
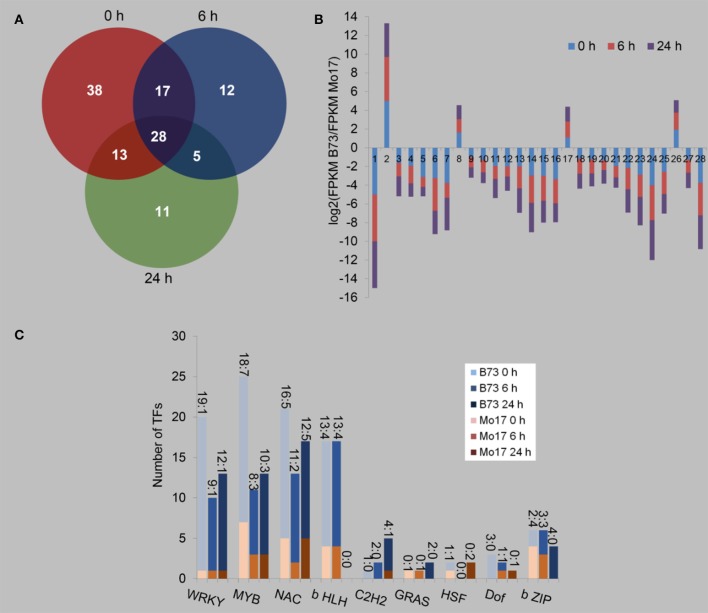
Differentially regulated transcription factors between B73 and Mo17 with and without *R. padi* treatment. The fourth maize leaves were each treated with 50 aphids, and samples were collected at 6 and 24 h (untreated leaves served as controls, depicted as 0 h). **(A)** Venn diagram indicating the number of differently expressed transcription factors (TFs) between B73 and Mo17 at each time point. **(B)** Expression profiles of the 28 common regulated TFs (X-axis represent randomly assigned gene numbers, and their gene IDs are in Table S7). **(C)** Distribution of the families of TF differentially regulated between B73 and Mo17, in control, at 6 and 24 h of *R. padi* herbivory-treated samples. On top of each bar, the number before the colon indicates the number of TFs that had greater expression in B73 than in Mo17, and the number after the colon indicates the number of TFs whose expression were lower in B73 than in Mo17. Full descriptions can be found in Table S7.

We next determined the transcript levels of TFs in B73 and Mo17 under control condition, 6 and 24 h after *R. padi* treatment (Figure 7, Table S7). Despite the large differences between the transcriptomes of B73 and Mo17, only 96 TFs were found to have different expression levels between B73 and Mo17 under the control condition, while 62 and 57 TFs were differentially expressed between these two lines after being treated with aphid feeding for 6 and 24 h, respectively (Figure 7A, Table S7); and among these, 38, 12, and 11 TF genes were specifically different only in the control samples and those induced by 6 and 24 h of *R. padi* herbivory. Only 28 TFs were differentially expressed between B73 and Mo17 at all time points, and these TFs showed the same patterns of regulation (Figure 7B, Table S7). The entirety of TFs that were differentially expressed between B73 and Mo17 were distributed in WRKY, MYB, NAC, b HLH, C2H2, GRAS, HSF, Dof, and b ZIP (Figure 7C, Table S7). Under all conditions, most TFs of the WRKY, MYB, NAC, b HLH, and C2H2 group had greater expression levels in B73 than in Mo17. Notably, the number of WRKY, MYB, and NAC TFs, whose transcript levels were higher in B73 than in Mo17, decreased after *R. padi* feeding; for example, in B73 0 h samples, 19 WRKYs had higher expressions than in Mo17, but in B73 6 and B73 24 h the number of TFs that had higher expression in B73 decreased to 9 and 12, respectively (Figure 7C). It is likely that TFs may have played an important role in shaping the differences of B73 and Mo17 transcriptomes under control and *R. padi*-induced conditions.

### Correlation between transcription factors and Bx biosynthesis genes

**Figure 8:**
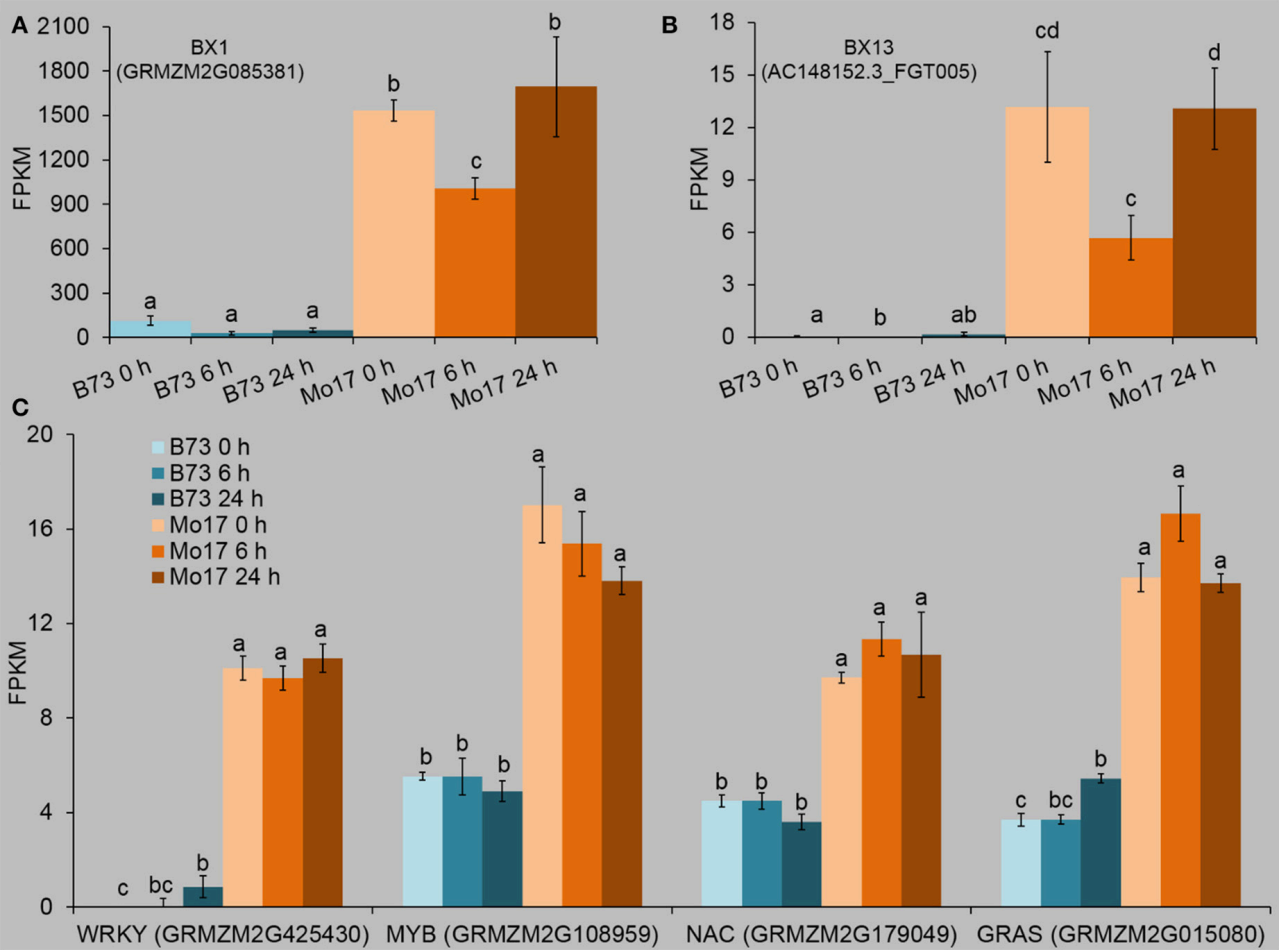
Expression profiles of Bx biosynthetic genes and transcription factors whose expression patterns were correlated with that of *BX1*. The fourth maize leaves were each treated with 50 aphids per plant, and samples were collected at 6 and 24 h after *R. padi* treatment (untreated leaves served as controls, depicted as 0 h). Relative changes of the transcript levels of *BX1* **(A)** and *BX13* **(B)**. Data were extracted from the FPKM values of the target genes in the RNA-seq datasets. **(C)** Transcription factors (TFs) which showed similar expression patterns to that of *BX1*. Values = means ± SE; *n* = 3; different letters above bars indicate significant differences (one-way ANOVA Duncan's multiple range test; *P* < 0.05). Full descriptions can be found in Table S7.

In order to understand the possible genetic basis for the different levels of Bxs in B73 and Mo17, the transcriptome data were screened for Bx biosynthesis-related genes that had been annotated (http://www.maizegdb.org/), and the newly reported *BX13* and *BX14* (Handrick et al. (2016). Most of the Bx biosynthetic genes had different expression levels between B73 and Mo17 (Table S7). Importantly, *BX1* and *BX13* levels were much higher in Mo17 than in B73 (at least 7.8 and 33 times greater in Mo17 than in B73) in all samples (Figures 8A,B, Table S7). *BX1* is very important for Bx biosynthesis (Butron et al., 2010); without the expression of *BX13*, DIM_2_BOA-Glc cannot be detected, suggesting *BX13* plays a significant role in Bx biosynthesis (Handrick et al., 2016). As Bx levels, such as DIM_2_BOA-Glc and HDMBOA-Glc, were much higher in Mo17 than in B73 (Figure 2A), *BX1* and *BX13* likely accounted for the high Bx contents in Mo17.

Only four TFs, a MYB (GRMZM2G108959), a GRAS (GRMZM2G015080), a NAC (GRMZM2G179049), and a WRKY (GRMZM2G425430), showed greater expression levels in Mo17 than in B73 under all conditions (Figure 8C, Table S7), suggesting that these four TFs may play a role in regulating the expression of *BX1* and *BX13*, resulting in the high Bx levels in Mo17.

### Differentially expressed volatile terpene biosynthesis genes

Terpene synthases (TPSs) catalyze the formation of diverse volatile terpenes (Bohlmann et al., 1998; Cheng et al., 2007; Dudareva et al., 2013). To investigate whether the volatile terpene biosynthesis pathways respond to *R. padi* feeding, we screened for the expression levels of 11 TPS genes in B73 and Mo17 that had been annotated by the Plant MetGenMAP to be 10 sesquiterpene synthases (TPS1 to 10) and one monoterpene synthase (GRMZM2G030583) (Joung et al., 2009). Among these 11 *TPS* genes inspected, *TPS8* (GRMZM2G038153) was induced (1.93- and 1.11-fold increased at 6 and 24 h, respectively) by *R. padi* infestation while *TPS10* was repressed (84 and 43% decreased after 6 and 24 h *R. padi* feeding, respectively) in B73, but not in Mo17 (Figure S2A, Table S8). *TPS2* (GRMZM2G046615) and *TPS3* (GRMZM2G064406) were 4.98- and 3.91-fold up-regulated by *R. padi* herbivory after 6 h *R. padi* feeding in Mo17, but not in B73 (Figure S2B, Table S8). We further compared the expression level of *TPS* genes between B73 and Mo17. Without *R. padi* feeding, there were five genes (*TPS2, TPS3, TPS7, TPS8*, and *TPS10*) differentially expressed between these two lines: *TPS2, TPS3, TPS8*, and *TPS10* showed higher levels in B73 compared to those in Mo17, while *TPS7* showed the opposite pattern (Figure S3A, Table S8). After 6 h of *R. padi* feeding, only *TPS7* and *TPS8* showed different expression levels between B73 and Mo17 (Figure S3B, Table S8), and after 24 h of *R. padi* feeding *TPS7* still expressed higher in B73 while the expression level of *TPS10* was lower in B73 than in Mo17 (Figure S3C, Table S8).

### Differentially spliced genes in B73 and Mo17

**Figure 9:**
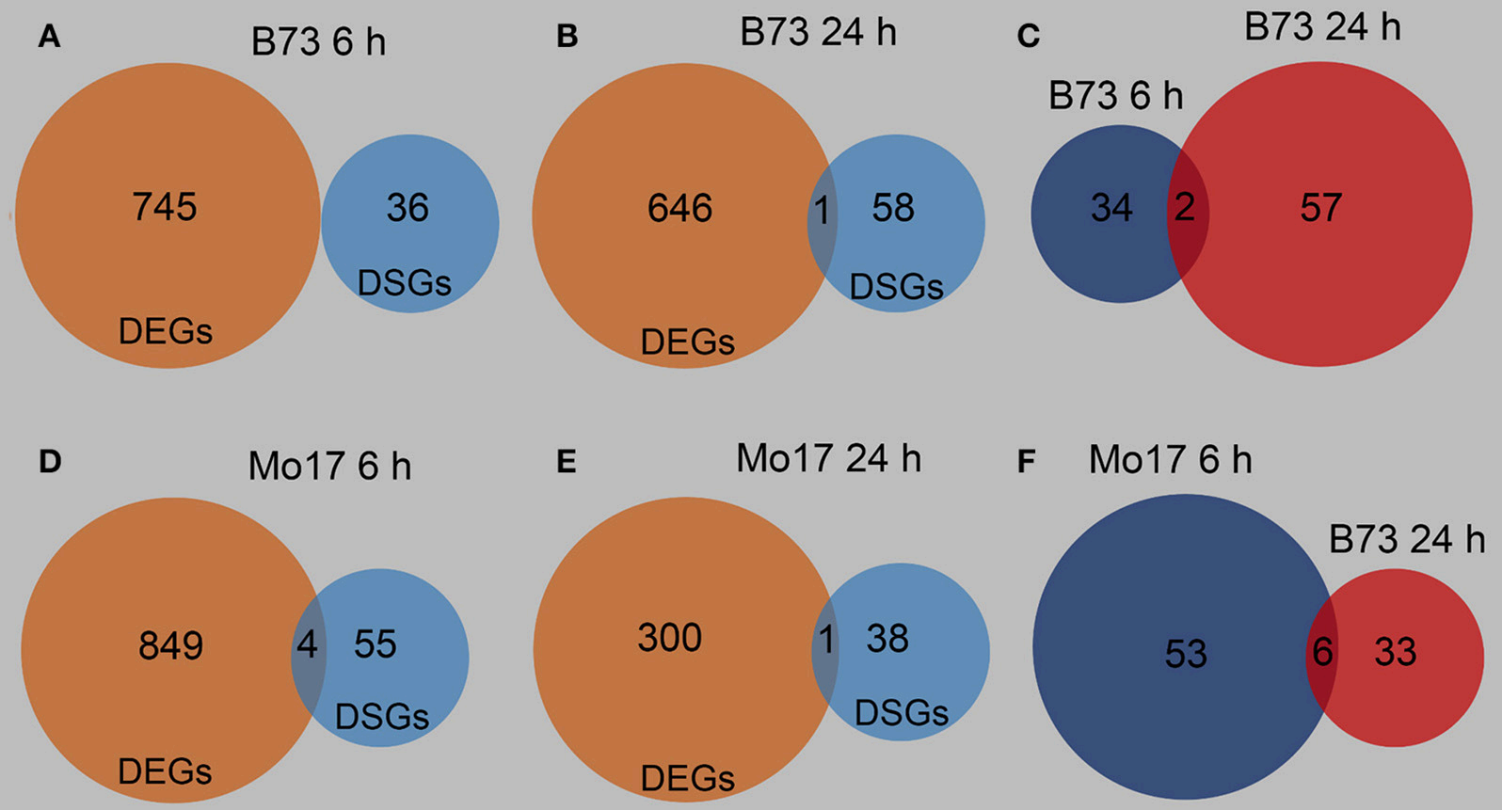
Overview of the differentially spliced genes in B73 and Mo17. The fourth maize leaves were each treated with 50 aphids per plant, and samples were collected at 6 and 24 h after *R. padi* treatment; untreated leaves served as controls. Venn diagrams indicating differentially expressed genes (DEGs) and differentially spliced genes (DSGs) at 6 h **(A)** and 24 h **(B)** of *R. padi* feeding in B73. Venn diagram showing specific and overlapping DSGs in B73 at 6 and 24 h of *R. padi* feeding **(C)**. Venn diagrams indicating DEGs and DSGs at 6 h **(D)** and 24 h **(E)** of *R. padi* feeding on Mo17. Venn diagram showing overlapping DSGs in Mo17 at 6 and 24 h of *R. padi* feeding **(F)**. Full descriptions can be found in Tables S3, S9.

The differentially spliced genes (DSGs) were investigated to determine whether AS might be involved in the response of B73 and Mo17 to *R. padi* feeding. 6 h after *R. padi* feeding on B73, 36 DSGs were identified, and there was no overlap between DEGs and DSGs at this time point (Figure 9A, Tables S3, S9); only one gene (among 59 DSGs) was both a DEG and a DSG after 24 h of *R. padi* feeding on B73 (Figure 9B, Tables S3, S9); among these 36 and 59 DSGs, only two genes showed changes in AS in B73 at both 6 and 24 h after *R. padi* feeding (Figure 9C, Table S9). Similarly, 59 and 39 DSGs were found in Mo17 after 6 and 24 h of *R. padi* feeding, respectively (Figure 9F, Table S9), and only a few genes were both regulated on the expression and AS level (Figures 9D,E, Tables S3, S9). Furthermore, six genes were found to be common between the DSGs identified in the samples of Mo17 6 and Mo17 24 h (Figure 9F, Table S9). To gain further insight into *R. padi*-induced dynamic AS responses in B73 and Mo17, pathway enrichment analysis was performed on the DSGs using MetGenMAP (Joung et al., 2009). No grouping of the DSGs in B73 6 and B73 24 h (Figure S4, Table S9) into enriched pathways showed no overlapping function. Notably, three pathways were in common among the enriched pathways in the DSGs of Mo17 6 and Mo17 24 h: synthesis of suberin, salicylate biosynthesis, and phenylpropanoid biosynthesis, and these are all defense-related (Figure S4, Table S9). These results indicate that B73 and Mo17 respond specifically to *R. padi* feeding on the AS level.

**Figure 10:**
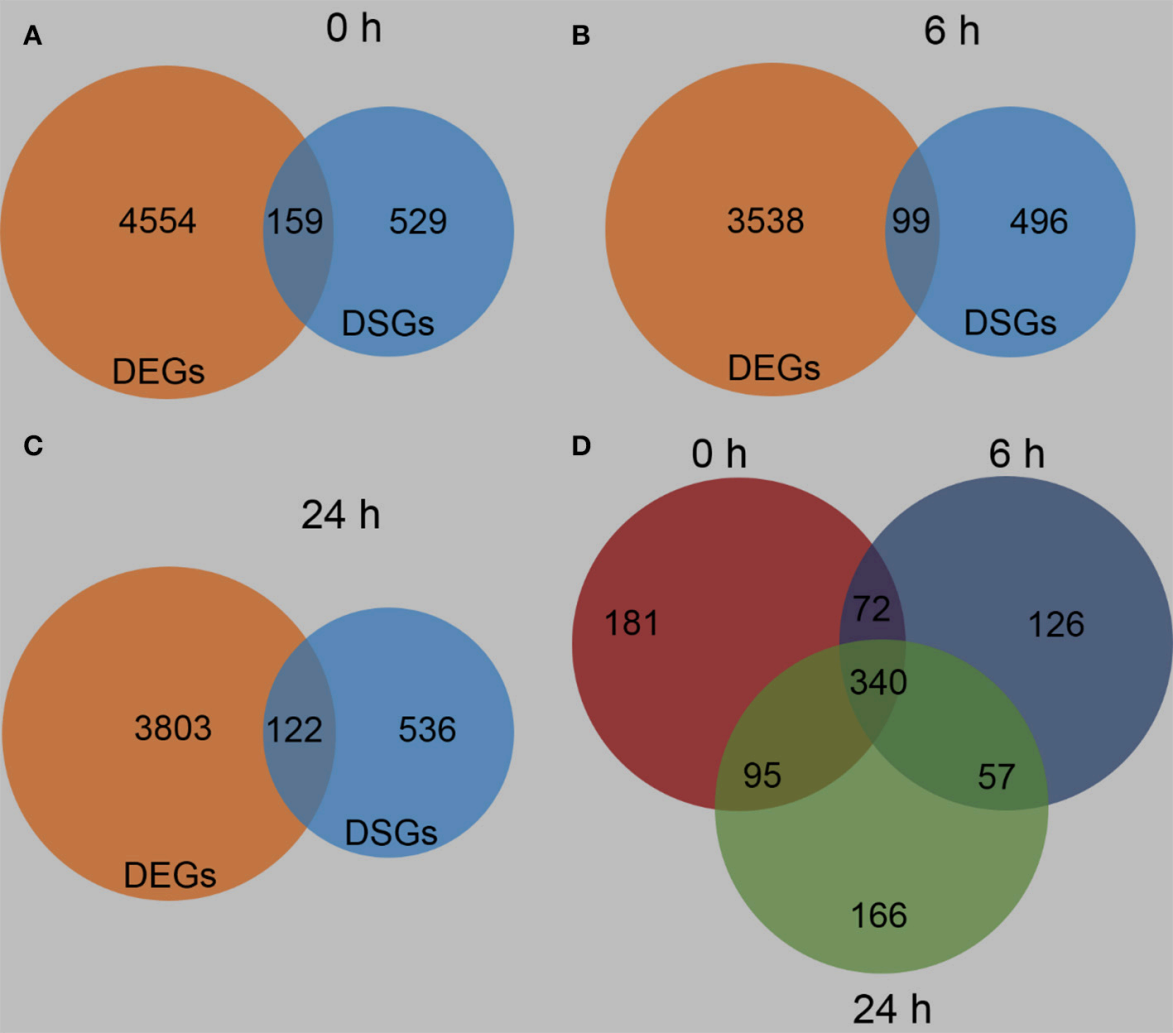
Differentially spliced genes between B73 and Mo17. The fourth maize leaves were each treated with 50 aphids, and samples were collected at 6 and 24 h (untreated leaves served as controls, depicted as 0 h). Venn diagrams indicating the differentially expressed genes (DEGs) and differentially spliced genes (DSGs) between B73 and Mo17 at 0 h **(A)**, 6 h **(B)**, and 24 h **(C)**. Venn diagram indicating specific and overlapping DSGs between B73 and Mo17 at 0, 6, and 24 h of *R. padi* feeding **(D)**. Full descriptions can be found in Tables S6, S10.

In order to gain further insight into the differences between these two maize lines, the DEGs and DSGs between B73 and Mo17 under control condition and at 6 and 24 h after *R. padi* treatment were determined. Consistent with the large differences in gene expression levels between these lines, 688 DSGs were identified between B73 0 and Mo17 0 h, and 159 genes were regulated both on expression and AS level (Figure 10A, Tables S6, S10). Similarly, after 6 and 24 h of *R. padi* infestation, B73 and Mo17 had 595 and 658 DSGs, respectively, and 99 and 122 genes were both DEGs and DSGs (Figures 10B,C, Tables S6, S10). The enriched pathways in the DSGs between B73 and Mo17 under control and after 6 h of *R. padi* feeding included flavin, spermine, and spermidine biosynthesis; terpenoid biosynthesis is the most enriched pathway in the DSGs identified between B73 and Mo17 after 24 h of *R. padi* herbivory (Figure S5, Table S10). Comparison of all these three groups of DSGs indicated that there were 181, 126, and 166 condition-specific DSGs between B73 and Mo17, while 340 genes were found consistently differentially spliced under all conditions (Figure 10D, Table S10). Furthermore, 10 TFs were found among these 340 DSGs, which belonged to the families of WRKY, bZIP, b HLH, GRAS, MYB, C2H2, MYB, and GATA (Table S10), and notably, none of these TFs showed differences in their expression levels between these two maize lines.

These results suggest that *R. padi* feeding induces specific AS patterns in different maize lines. Furthermore, AS difference also existed between B73 and Mo17 and is largely independent of the regulation of gene expression levels.

## Discussion

Within a species, phenotypic variations caused by heritable genetic diversity are the driving force in adaptation to the ever-changing environment. Like many other traits, phenotypic variations of resistance to insects has been demonstrated to vary among populations of wild plants (Wu et al., 2008) and varieties of crops (Meihls et al., 2013; Wang et al., 2014; Tzin et al., 2015b). Crop plants, such as maize, have rich resources of genetic diversity due to thousands of years of artificial and natural selection. These diverse genetic crop resources are not only important for breeding new varieties with improved agronomic and resistance traits, but are also the material for scientists studying the mechanisms underlying phenotypic variations.

**Figure 11:**
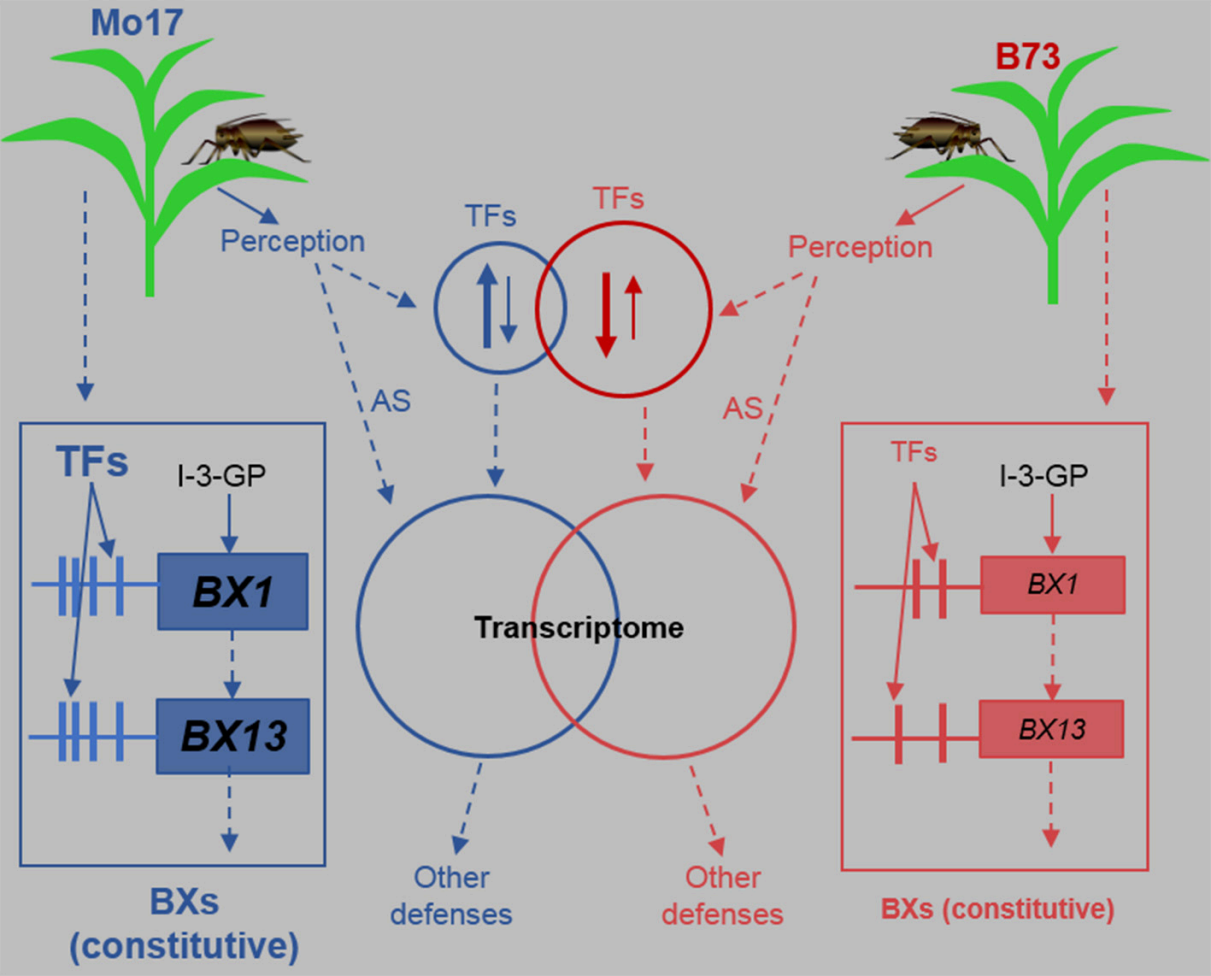
A model summarizing the defense responses of B73 and Mo17 against the aphid *R. padi*. Maize plants respond to *R. padi* feeding mainly by perceiving certain aphid-derived elicitors through unknown receptors, thereby transcriptionally up- or down-regulating TFs (transcription factors). In B73, more TFs are regulated than in Mo17 and most TFs are down-regulated, while in Mo17 most TFs are up-regulated. TFs and other pathways, including alternative splicing (AS), continuously shape the transcriptomes of B73 and Mo17, resulting in changes in defenses. Importantly, certain TFs, that are not regulated by *R. padi* herbivory, target the promoters of *BX1* and *BX13* and have higher activity in Mo17 than in B73, resulting in much higher expression levels of *BX1* and *BX13* and thus high contents of benzoxazinoids (Bxs) in Mo17. The promoters of *BX1* and *BX13* in Mo17 may also possess more *cis*-elements that allow more efficient transcription. I-3-GP: indole-3-glycerol phosphate; Red and blue arrows and circles represent B73 and Mo17, respectively. Up and down arrows in the circles indicate up-regulation or down-regulation, and the thickness of these arrows represent the numbers of genes. The sizes of the circles indicate the number of regulated TF genes and the size of letters represent activity, expression, or concentrations. Vertical lines connected with the boxes of *BX1* and *BX13* represent their promoters, and the horizontal bars symbolize *cis*-elements. Bxs are a group of metabolites produced by BX1 to BX14.

Many lines of evidence have indicated the importance of Bxs defense against insects. In maize, Bx levels are negatively correlated with the growth of the grain aphid *Sitobion avenae* and *R. padi* (Thackray et al., 1990), supplementation of Bxs to artificial diet increased the mortality of the greenbug *Schizaphis graminum* (Argandona et al., 1983), and Bxs are also important for the defense against the European Corn Borer *Ostrinia nubilalis* (Niemeyer, 2009). Our detailed analyses in control and *R. padi*-induced B73 and Mo17 indicate that Bx biosynthesis-related genes and Bx accumulation are not regulated by *R. padi* feeding. This is consistent with the finding that *R. maidis* herbivory on B73 did not change the concentrations of HMBOA-Glc, DIM_2_BOA-Glc, and HDMBOA-Glc (Meihls et al., 2013), although *BX1, BX2, BX6*, and *BX7* showed transient increases (Tzin et al., 2015a). We found that the transcript levels of *BX1* and *BX13* are substantially greater in Mo17 than in B73. BX1 is the initial enzyme in the Bx biosynthesis pathway, and genetic analysis suggested that the high expression level of the *BX1* in Mo17 very likely accounts for its abundant Bx content (Zheng et al., 2015). Recently, Handrick et al. (2016) identified *BX13* as a *2-oxoglutarate-dependent dioxygenase*, encoding an enzyme that converts DIMBOA-Glc to TRIMBOA-Glc, and maize lines having a mutation in BX13 displayed compromised resistance to the leaf aphid *R. maidis*. Therefore, the high expression levels of *BX1* and *BX13* in Mo17 are very likely the reason for its high contents of Bxs and thus high resistance to *R. padi* (Figure 11). The *cis-* and *trans*-elements of *BX1* and *BX13*, such as TFs and TF-binding sites in the promoters, need to be studied to reveal the underlying mechanism by which these plants maintain their high expression of these two BX-biosynthetic genes (Figure 11).

In contrast to Bxs, most detected phenolic and flavonoid metabolites showed higher contents in B73 than in Mo17. Wheat cultivars with high concentrations of soluble and cell wall-bound phenolics were much less attractive to *R. padi* than the cultivars with low phenolic concentrations (Leszczynski et al., 1985), and another study showed that phenolics in triticale hybrids were negatively associated with the attractiveness to *R. padi* (Wójcicka, 2010). Notably, *R. padi* aphid feeding led to decrease of phenolics in B73, but had no effect on most phenolics in Mo17. More studies are needed to clarify whether phenolics are involved in the defense against *R. padi* in maize.

Tzin et al. (2015a) found that on the maize *tps2/3::Ds* mutant, *R. maidis* reproduction was decreased by ~73% compared to the reproduction of aphids on the wild-type W22, suggesting that the terpene products of TPS2 and TPS3 may promote the growth of aphids as feeding stimulants (Tzin et al., 2015a). We found that *TPS2* and *TPS3* showed higher expression levels in B73 than in Mo17 under normal condition (Figure S3A, Table S8). Thus, it is possible that B73 contains greater levels of TPS2/3-produced feeding stimulants (terpenes) than does Mo17, which partly accounts for the susceptibility of B73 to aphids. TPS10 catalyzes the formation of (*E*)-β-farnesene, (*E*)-α-bergamotene, and sesquiterpene hydrocarbons, and overexpression of *TPS10* protect plants by attracting the parasitoid (*C. marginiventris*) of lepidopteran insects (Schnee et al., 2006). Even though *TPS10* expression was suppressed by *R. padi* feeding in B73 (not in Mo17), B73 always exhibited greater levels of *TPS10* than did Mo17 (Figure S3, Table S8), suggesting that B73 may have better indirect defenses than does Mo17. Whether the terpene products of TPS10 may also act as *R. padi* feeding stimulants remains unknown. Moreover, *TPS7*, which participates in sesquiterpene biosynthesis, consistently showed higher levels in Mo17 than in B73 (Figure S3, Table S8), and this may also partly contribute to the different resistance to *R. padi* in these two maize lines. Further analysis of the contents of terpenes in different maize lines and the functions of individual terpenes in attracting the aphid predators (such as parasitoids) or stimulating/deterring aphid feeding are needed for further understanding the correlation between the differentially expressed *TPS*s and cultivaral differences of aphid resistance.

A recent transcriptomic study on *R. maidis*-induced B73 allows to compare the responses of B73 to *R. maidis* (Tzin et al., 2015a) and *R. padi* (this study). Most of the significantly changed transcripts after *R. maidis* feeding were up-regulated (Tzin et al., 2015a), whereas *R. padi* herbivory transcriptionally suppressed rather than induced genes. More than 1,600 transcripts, whose expression profile changed at least at one time-point after *R. maidis* feeding clustered into six groups; the biological processes included the shikimate pathway, biosynthesis of aromatic amino acids, salicylic acid, jasmonic acid, auxin, and ethylene biosynthesis from Met. More than 1,100 genes induced by *R. padi* feeding were clustered to three groups, whose biological processes included chlorogenic acid biosynthesis I and chlorogenic acid biosynthesis II. In parallel to the transcriptomic profiles, *R. maidis* feeding up-regulated most of the metabolites in B73, but our data showed that most detected metabolites in *R. padi*-infested B73 did not change significantly (Figure 2, Table S1). Thus, similar to *Miscanthus sinensis*, which was found to be resistant to *R. padi* but susceptible to *R. maidis* (Huggett et al., 1999), B73 responded distinctive to *R. maidis* and *R. padi*, and this was likely because of the different aphid species used, but could also be related to different treatments (10 adult *R. maidis* and 50 adult *R. padi*) or plant growth stages (2- and 3-week-old seedlings, respectively).

B73 and Mo17 responded to *R. padi* feeding with high specificity on the transcriptomic level; For instance, most of the TFs in B73 were down-regulated by *R. padi* herbivory, while most of those in Mo17 were up-regulated (Figure 11). Importantly, these large transcriptomic differences were independent of *R. padi* feeding. In line with this, we found more differentially expressed TF between B73 and Mo17 under any condition than there were differentially expressed TF induced by *R. padi* herbivory in these lines. All these data are consistent with the large genetic differences between maize inbreed lines (Springer et al., 2009; Jiao et al., 2012). Although thousands of genes were differentially expressed between B73 and Mo17, only a small number (at most 96 in the control samples) were TFs. It is likely that at least some of these TFs, if not all, accounted for the transcriptomic differences. Using genetic mapping and allele-specific transcript measurements, Zheng et al. (2015) found that both *cis*-element(s) and *trans*-acting factors affect *BX1* transcript levels in Mo17. Our analysis indicated that only four differentially expressed TFs, a WRKY, a MYB, a NAC, and a GRAS gene, showed a good correlation with the *BX1* expression patterns in B73 and Mo17 (Figure 8, Table S7). Moreover, emerging evidence has pointed to the role of AS in regulating TF activity (Seo et al., 2013). In addition to these four TFs that have constantly different expression levels between B73 and Mo17, 10 other TFs were found to be differentially spliced between B73 and Mo17 under all conditions (Table S10). It is possible that, among these 14 differentially expressed or spliced TFs, some influence the expression of *BX1* and further affect the content of Bxs. Functional studies focusing on these TFs are needed to elucidate whether they play a role in regulating Bx biosynthesis.

Alternative splicing (AS) is highly prevalent in plants, and is known to rapidly change the transcriptome and proteome diversity by altering transcription complexity in response to stresses (Gassmann, 2008; Syed et al., 2012; Reddy et al., 2013; Yang et al., 2014). Our comparison of DEGs and DSGs between B73 and Mo17 under control, 6 and 24 h of aphid herbivory indicated that only very few genes were regulated on both expression and AS level (Figures 10A–C, Tables S6, S10). It is likely that AS and gene expression are regulated largely independent in maize response to aphid feeding. This is consistent with a study on AS events in the response of *N. attenuata* to the chewing caterpillar *M. sexta*, where 5 h of *M. sexta* feeding induced 180 and 356 DSGs in leaves and roots, respectively, and there was only little overlap (31 genes in leaves and six genes in roots) between differentially expressed and DSGs in both leaves and roots (Ling et al., 2015). After *R. padi* feeding, B73 and Mo17 had only some genes that were DSGs at both 6 and 24 h (Figures 9C,F, Table S9), indicating a highly time-specific regulation of AS. The finding that B73 and Mo17 have many specific DSGs under control and *R. padi*-feeding conditions (Figure 10D, Table S10) also suggest an important role of AS in diversifying the specific responses to *R. padi* infestation on different maize lines. A growing body of evidence has revealed that AS influences gene functions in plants. For example, the FLK protein influences flowering time in *Arabidopsis*, and FLK in prmt5 mutants (impaired in AS regulation) cannot be normally spliced, resulting in a late flowering phenotype (Pei et al., 2007; Deng et al., 2010); PRR9 is a core-clock gene, which influences the circadian rhythm in *Arabidopsis*, and altered splicing of PRR9 caused an impairment of the circadian clock (Sanchez et al., 2010); JAZ proteins play an important role in repressing jasmonic acid signaling. AS of JAZ10 produces different splice variants, and JA-induced JAZ10.4 protein accumulation has been suggested to be an important feedback regulator to mediate JA signal desensitization (Moreno et al., 2013). Further functional studies on the different spliced isoforms of alternatively spliced genes are needed to understand if and how AS regulates maize responses to aphid herbivory.

Taken together, our large-scale data analyses revealed highly distinct metabolic and transcriptomic features in B73 and Mo17, and the differences remained large after aphid *R. padi* feeding (Figure 11). Furthermore, we identified a group of TFs that might contribute to the different resistance to *R. padi* by regulating the accumulation of Bxs. These datasets could be used for further dissecting the genetic basis for maize defense against aphids and for breeding new maize varieties with enhanced aphid resistance.

## Author contributions

JW conceived and designed the study. JQ, JL, and CH provided advice on the experimental design. SW and JS performed phenotype measurements. JS, HL, HZ, CZ, and YX analyzed data. JS and JW wrote the manuscript. All authors reviewed and edited the manuscript.

### Conflict of interest statement

The authors declare that the research was conducted in the absence of any commercial or financial relationships that could be construed as a potential conflict of interest.
