# Supplementary material for: Transcriptomics and Alternative Splicing Analyses Reveal Large Differences between Maize Lines B73 and Mo17 in Response to Aphid Rhopalosiphum padi Infestation
Source: Front Plant Sci. 2017 Oct 10;8:1738. doi: 10.3389/fpls.2017.01738 (PMC5641392; doi:10.3389/fpls.2017.01738)
Supplement: Supplementary file 2 [file Table2.DOCX]

**Table S2.** Numbers of the differentially expressed genes in the response of B73 and Mo17 to *R. padi* feeding

| Samples | Different expressed genes (*P* < 0.05) | Up-regulated genes  (*P* < 0.05) | Down-regulated genes (*P* < 0.05) |
| --- | --- | --- | --- |
| B73 6 h | 3789 | 1800 | 1989 |
| B73 24 h | 3420 | 1645 | 1775 |
| Mo17 6 h | 4127 | 2221 | 1906 |
| Mo17 24 h | 1786 | 962 | 824 |

Note: Complete datasets can be found in Table S3.
