# Supplementary material for: Transcriptomics and Alternative Splicing Analyses Reveal Large Differences between Maize Lines B73 and Mo17 in Response to Aphid Rhopalosiphum padi Infestation
Source: Front Plant Sci. 2017 Oct 10;8:1738. doi: 10.3389/fpls.2017.01738 (PMC5641392; doi:10.3389/fpls.2017.01738)
Supplement: Supplementary file 11 [file DataSheet1.pdf]

Supplementary Material

Transcriptomics and alternative splicing analyses reveal large differences between maize lines B73 and Mo17 in response to aphid *Rhopalosiphum padi* infestation

Juan Song, Hui Liu, Huifu Zhuang, Chunxia Zhao, Yuxing Xu, Shibo Wu, Jinfeng Qi, Jing Li, Christian Hettenhausen*, and Jianqiang Wu*

***Corresponding authors**

Jianqiang Wu and Christian Hettenhausen

Email: wujianqiang@mail.kib.ac.cn; hettenhausen@mail.kib.ac.cn

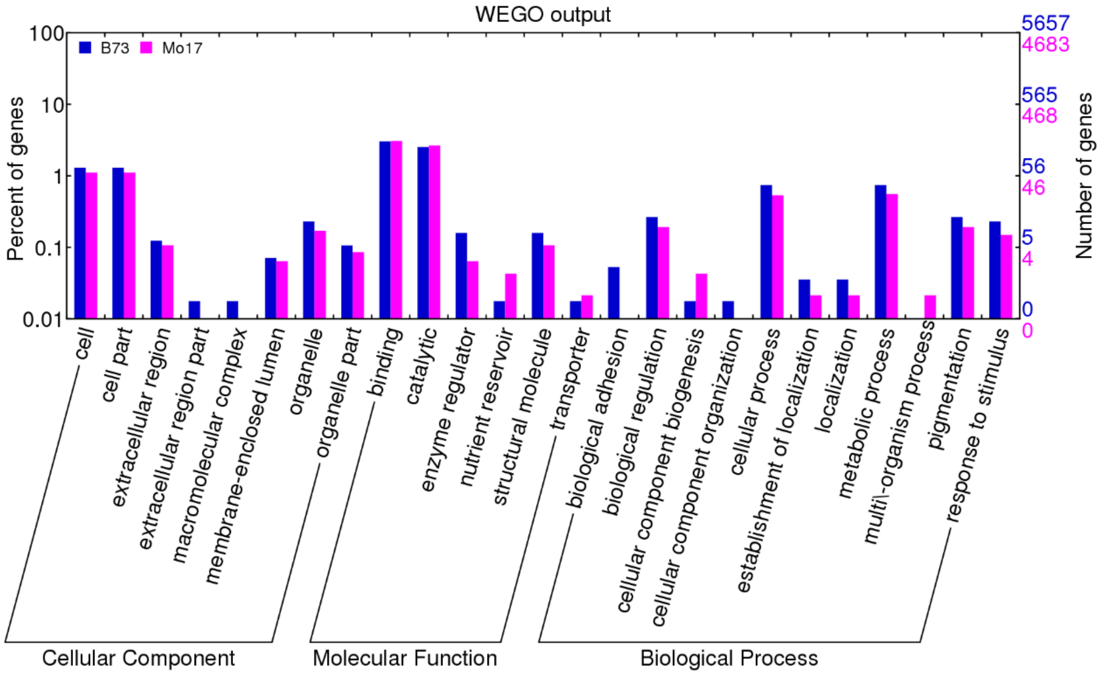

Figure S1. Functional analysis of *R. paid* herbivory-regulated genes in B73 and Mo17 based on RNA-Seq data.

GO functional enrichment analysis of differentially expressed genes (DEGs) in B73 and Mo17 after *R. padi* herbivory. Based on sequence homology, 7931 DEGs are summarized in three main categories: cellular component, molecular function and biological process. The X-axis expresses the definition of GO terms. The right Y-axis indicates the number of genes in each category. The left Y-axis indicates the percentage of targeted genes mapped by the GO terms. Complete data can be found in Table S3.

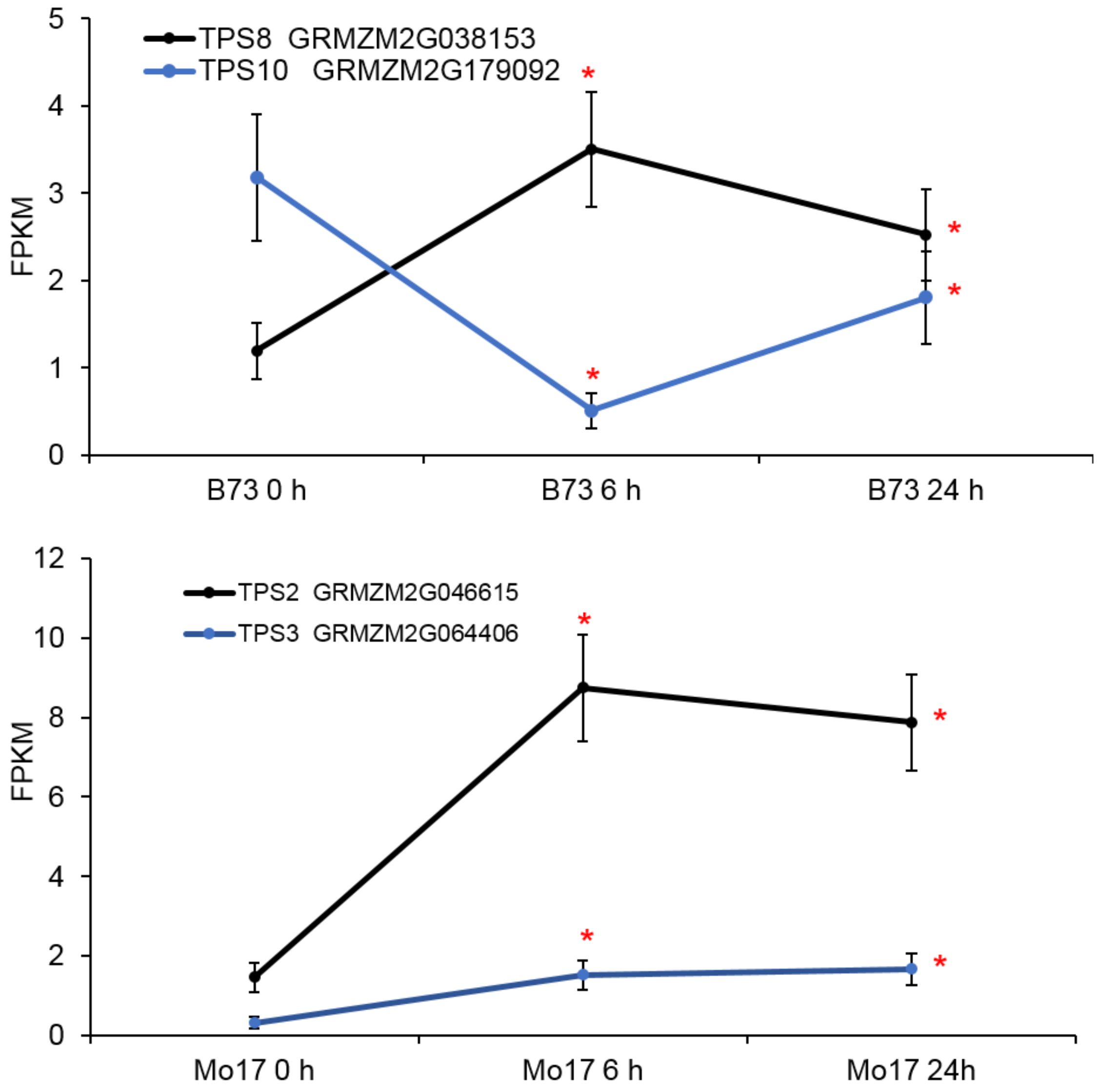

Figure S2. Differentially expressed terpene biosynthesis genes in B73 and Mo17, in response to *R. padi* feeding.

Maize B73 and Mo17 leaves were treated with 50 aphids per plant, and samples were collected at 6 and 24 h after *R. padi* treatment (untreated samples served as controls, depicted as 0 h). Differentially expressed genes (DEGs) in terpene biosynthesis pathways after 6 and 24 h of *R. padi* feeding in B73 **(A)** and Mo17 **(B)** are shown. Complete data can be found in Table S8.

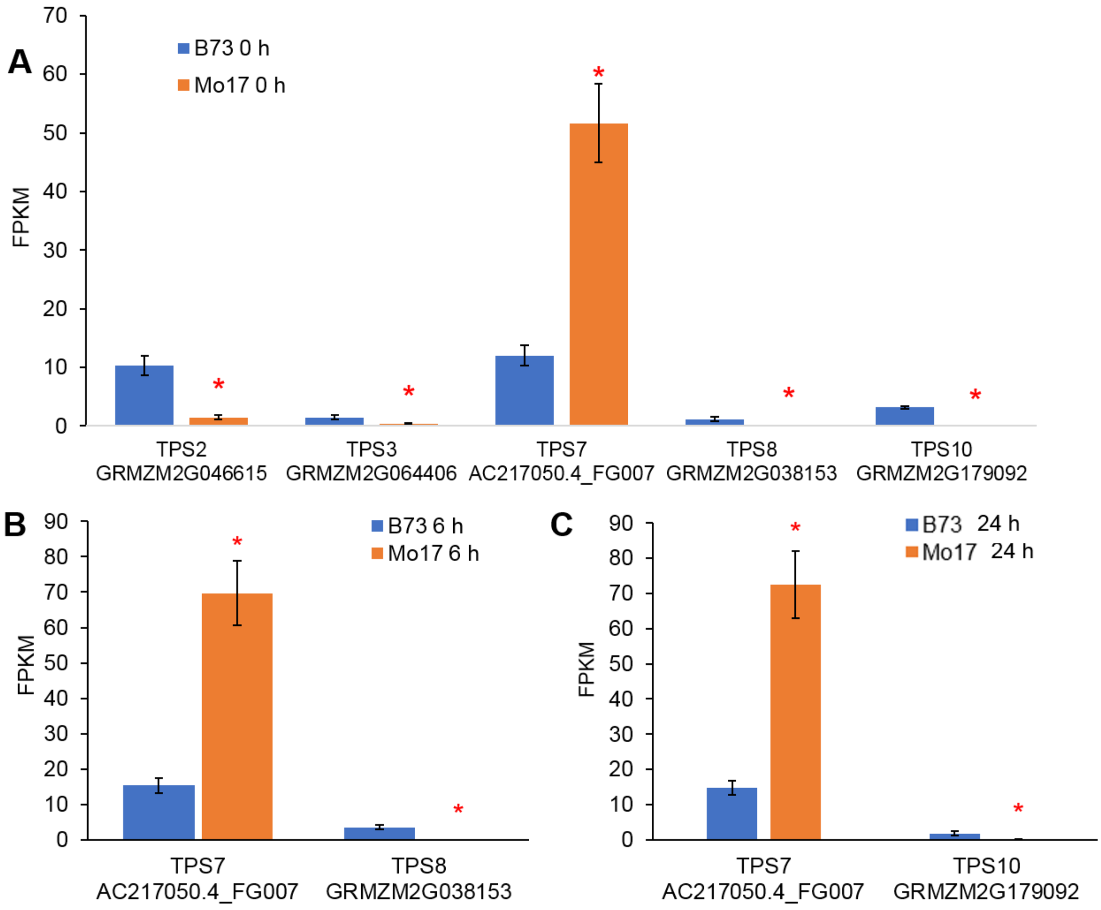

Figure S3. Differentially expressed terpene biosynthesis genes between B73 and Mo17 with and without *R. padi* treatment.

Maize B73 and Mo17 leaves were treated with 50 aphids per plant, and samples were collected at 6 and 24 h after *R. padi* treatment (untreated samples served as controls, depicted as 0 h). DEGs in terpene biosynthesis pathways between B73 and Mo17 without *R. padi* feeding **(A)** and after 6 **(B)** and 24 h **(C)** of *R. padi* feeding. Complete data can be found in Table S8.

**Pathway name (DSGs B73 6 h)**	***P- value***
GDP-mannose biosynthesis I	1.05E-02
Homoserine biosynthesis	1.05E-02
GDP-mannose biosynthesis II	1.05E-02
Ascorbate biosynthesis I (L-galactose pathway)	1.29E-02
GDP-mannose metabolism	1.45E-02
Lysine biosynthesis VI	2.09E-02

**Pathway name (DSGs B73 24 h)**	***P- value***
Anandamide degradation	1.29E-02
Methanol oxidation to formaldehyde	1.92E-02
Xanthophyll cycle	2.24E-02
Antheraxanthin and violaxanthin biosynthesis	2.24E-02
Sulfate activation for sulfonation	3.50E-02

**Pathway name (DSGs Mo17 6 h)**	***P- value***
Suberin biosynthesis	1.67E-03
Asparagine biosynthesis I	1.92E-02
Superpathway of asparagine biosynthesis	1.92E-02
Asparagine biosynthesis II	1.92E-02
Salicylate biosynthesis	3.58E-02
Phenylpropanoid biosynthesis, initial reactions	4.29E-02

**Pathway name (DSGs Mo17 24 h)**	***P- value***
Myo-inositol biosynthesis	1.62E-03
Salicylate biosynthesis	2.40E-02
Phenylpropanoid biosynthesis, initial reactions	2.88E-02
Glycerol degradation I	3.67E-02
Lactose degradation III	3.82E-02
Suberin biosynthesis	4.29E-02

Figure S4. The pathways enriched in the differentially spliced genes in response to *R. padi* infestation in B73 and Mo17.

Maize B73 and Mo17 leaves were treated with 50 aphids per plant, and samples were collected at 6 and 24 h after *R. padi* treatment (untreated samples served as controls). MetGenMAP was used for pathway enrichment analysis to identify metabolic functions of the differentially spliced genes (DSGs) in response to *R. padi* infestation. Detailed data can be found in Table S9.

**Pathway name (DSGs 0 h)**	***P- value***
Flavin biosynthesis	1.47E-02
Spermine biosynthesis	2.04E-02
Coniferin metabolism	3.44E-02
Spermidine biosynthesis	3.78E-02
Aerobic respiration -- electron donor II	4.62E-02

**Pathway name (DSGs 6 h)**	***P- value***
Flavin biosynthesis	7.30E-03
Spermine biosynthesis	1.02E-02
Spermidine biosynthesis	1.93E-02
Acrylonitrile degradation	2.41E-02
Aldoxime degradation	2.41E-02
Superpathway of polyamine biosynthesis II	3.07E-02
Cyanide detoxification	3.59E-02
IAA biosynthesis IV	3.59E-02
1,4-dihydroxy-2-naphthoate biosynthesis II (plants)	4.76E-02

**Pathway name (DSGs 24 h)**	***P- value***
Flavin biosynthesis	1.47E-02
Octaprenyl diphosphate biosynthesis	2.04E-02
All trans undecaprenyl diphosphate biosynthesis	2.04E-02
Nonaprenyl diphosphate biosynthesis	2.04E-02
Dodecaprenyl diphosphate biosynthesis	2.04E-02
Hexaprenyl diphosphate biosynthesis	2.04E-02
Decaprenyl diphosphate biosynthesis	2.04E-02
Geranylgeranyldiphosphate biosynthesis	2.04E-02
Heptaprenyl diphosphate biosynthesis	2.04E-02
Tridecaprenyl diphosphate biosynthesis	2.04E-02
Acrylonitrile degradation	3.44E-02
Aldoxime degradation	3.44E-02
Superpathway of geranylgeranyldiphosphate biosynthesis II (via MEP)	4.17E-02
Superpathway of geranylgeranyldiphosphate biosynthesis I (via mevalonate)	4.41E-02

Figure S5. Overview of differentially spliced genes between B73 and Mo17.

Maize B73 and Mo17 leaves were treated with 50 aphids per plant, and samples were collected at 6 and 24 h after *R. padi* treatment (untreated samples served as controls, depicted as 0 h). MetGenMAP was used for pathway enrichment analysis to identify metabolic functions of the differentially spliced genes (DSGs) between B73 and Mo17 under control, 6 h, and 24 h- *R. padi* treatment. Complete data can be found in Table S10.
